# Anchoring of Polymer Loops on Enzyme-Immobilized Mesoporous ZIF-8 Enhances the Recognition Selectivity of Angiotensin-Converting Enzyme Inhibitory Peptides

**DOI:** 10.3390/molecules28073117

**Published:** 2023-03-31

**Authors:** Zefen Wang, Qian Zhou, Siyuan Liu, Dankui Liao, Pengru Liu, Xiongdiao Lan

**Affiliations:** 1 School of Chemistry and Chemical Engineering, Guangxi University, Nanning 530004, China; 2 Institute of Biological Manufacturing Technology Co., Ltd., Guangxi Institute of Industrial Technology, Nanning 530002, China; 3 Guangxi Key Laboratory for Polysaccharide Materials and Modifications, Guangxi Minzu University, Nanning 530006, China; 4 Key Laboratory of New Technology for Chemical and Biological Transformation Process of Guangxi Higher Education Institutes, Guangxi Minzu University, Nanning 530006, China

**Keywords:** polymer loops, angiotensin converting enzyme, immobilization of enzyme, mesoporous ZIF-8, recognition selectivity

## Abstract

Immobilized angiotensin-converting enzyme (ACE) is a promising material for the rapid screening of antihypertensive drugs, but the nonspecific adsorption is a serious problem in separation processes involving complex biological products. In this study, triblock copolymers with dopamine (DA) block as anchors and PEG block as the main body (DA-PEGx-DA) were attached to an immobilized ACE (ACE@mZIF-8/PDA, AmZP) surface via the “grafting to” strategy which endowed them with anti-nonspecific adsorption. The influence of DA-PEGx-DA chain length on nonspecific adsorption was confirmed. The excellent specificity and reusability of the obtained ACE@mZIF-8/PDA/DA-PEG_5000_-DA (AmZPP_5000_) was validated by screening two known ACE inhibitory peptides Val-Pro-Pro (VPP, competitive inhibitory peptides of ACE) and Gly-Met-Lys-Cys-Ala-Phe (GF-6, noncompetitive inhibitory peptides of ACE) from a mixture containing active and inactive compounds. These results demonstrate that anchored polymer loops are effective for high-recognition selectivity and AmZPP_5000_ is a promising compound for the efficient separation of ACE inhibitors in biological samples.

## 1. Introduction

Recognized as a potential target for hypertension treatment, the angiotensin-converting enzyme (ACE) is the key enzyme for blood pressure regulation and is mainly localized on the membranes of lungs, heart, kidney, and intestinal cells [1,2,3]. The functional balance of ACE is crucial to blood pressure regulation. High ACE activity promotes angiotensin II production and bradykinin hydrolysis, leading to vasoconstriction and increased blood pressure [4,5]. Several chemical synthetic ACE inhibitors, such as captopril, enalaprilat, temocapril and lisinopril, have been used in hypertension treatment, but most of them produce side effects [6,7,8]. Thus, novel ACE inhibitors for developing anti-hypertension drugs are needed.

In recent years, numerous naturally-derived ACE inhibitory peptides have been reported to possess a strong inhibitory effect on ACE and have no negative side effects [9,10,11]. However, the screening of ACE inhibitory peptides from complex mixtures of natural products through traditional separation technologies is tedious and time-consuming. Thus, rapid screening methods for discovering ACE inhibitory peptides for novel hypertension therapeutics are in high demand.

Immobilized enzymes have been developed as affinity separation media for selectively enriching bioactive compounds from complex mixtures [12,13,14,15]. In recent decades, a number of materials, including magnetic beads [16,17,18], microspheres [19,20,21], capillary electrophoresis [22], and MOFs [23], have been used to immobilize ACE on surfaces and screen ACE inhibitory peptides from protein hydrolysate. However, these immobilized enzymes have a drawback: the natural conformation of an enzyme cannot be maintained during the separation process [24,25,26]. Recent studies on enzyme immobilization have demonstrated that enzymes can be encapsulated in situ in MOFs and retain the stability of their conformations [27,28,29]. However, MOFs-encapsulated enzymes have strong nonspecific adsorption for MOFs and thus show false selectivity [30,31]. Thus, anti-nonspecific protein adsorption is one of the main challenges in designing novel affinity separation media.

A common and promising method for solving this problem is surface modification with inert hydrophilic polymers [32,33,34]. Polymer constituents and morphology are important in this method. Polyethylene glycol (PEG) is one of the popular and most suitable polymer materials surface modification and considered the “gold standard” antifouling and biocompatible polymer by the FDA [35,36,37]. The highly-hydrated PEG chains on the surfaces of solids can effectively reduce nonspecific protein absorption. Most polymer chains are tethered through one end group to a surface. Loop-type polymers can be obtained by anchoring the chain ends of linear polymers to surfaces. Compared with linear polymers, loop-type polymers can better resist nonspecific protein adsorption [38,39,40]. In addition, introducing dopamine (DA) to the ends of polymers facilitates polymer surface grafting due to universal attachment and the ease of use of catechol groups [41].

In this work, ACE was encapsulated in situ in mesoporous ZIF-8 for the preparation of AmZP. A novel immobilized ACE AmZPP_x_ (x = molecular weight of PEG) was obtained by anchoring an ABA triblock copolymer DA-PEG_x_-DA with block A as a catechol anchor attracted to surfaces and block B in which PEG is a functional block on AmZP surfaces. The developed materials were systematically characterized. Two main inhibitory types of ACE inhibitory peptides are known: competitive and noncompetitive inhibition. The casein-derived peptide Val-Pro-Pro (VPP) is one of the ACE inhibitory peptides with competitive inhibition [42,43] and has been added to milk as a food additive. In our previous research, Gly-Met-Lys-Cys-Ala-Phe (GF-6) was isolated from *Saurida elongata* and acted as a non-competitive inhibitor to ACE [44,45]. Thus, VPP and GF-6 were applied to investigate the adsorption performance, selectivity, separation ability, and reusability of AmZPP_x_. This work is the first to apply loop-polymer-modified immobilized enzymes to bioactive compounds screening.

## 2. Results and Discussion

### 2.1. Synthesis of AmZPP_x_

**Figure 1:**
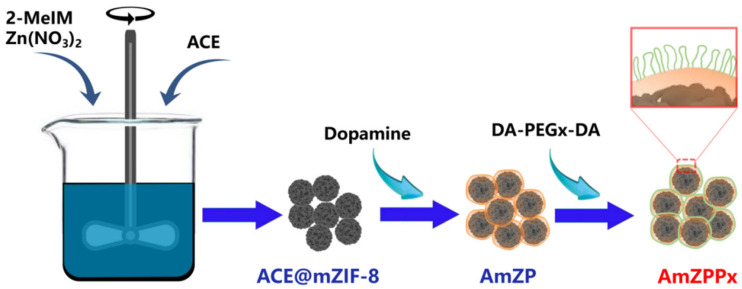
The synthesis procedure of AmZPP_x_.

Highly selective materials for ACE inhibitory peptide screening were prepared using a polymer DA-assisted surface-modified ACE–MOF composite. The synthesis process is shown in Figure 1. First, ACE was encapsulated in situ in mZIF-8, approximately 90% of the enzyme was incorporated and the loading of protein was 0.11 mg/mg composites. Second, ACE@mZIF-8 was added to a DA solution water to form a PDA coating layer. This special structure of the AmZP afforded the materials low enzyme leakage [46]. Then, AmZP was added to a polymer solution for surface modification and reduction of nonspecific adsorption. 

**Figure 2:**
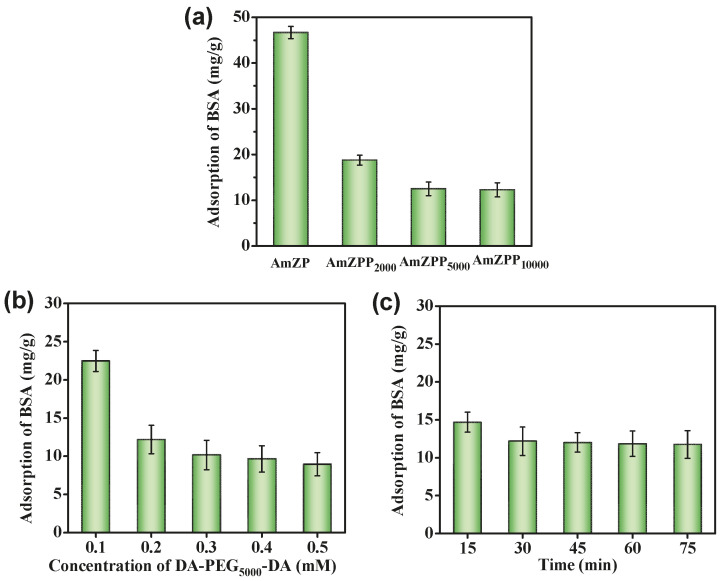
The BSA adsorption performance of AmZPP_x_ with the different polymer length (**a**), concentration of DA-PEG_5000_-DA (**b**) and contact time (**c**).

In this work, Bovine Serum Albumin (BSA) was used as a nonspecific protein model because it can easily attack many materials through nonspecific interactions [47]. Figure 2a shows the effect of the length of the PEG midblock on anti-nonspecific adsorption. The adsorption capacity of AmZPPx on BSA was significantly lower than of AmZP because of the repulsive force caused by the steric hindrance of loop-polymer-coated surfaces [40]. In addition, the BSA adsorption decreased with the increasing molecular weight of PEG because of the weak antiprotein effect when the polymer chain length was short. However, when the molecular weight of the PEG midblock increased to 5000, the effect on the adsorption of BSA was quite small. This result was consistent with that of a previous report [41]. Therefore, AmZPP_5000_ was employed in subsequent studies. The effects of the dosages of DA-PEG_5000_-DA and modification time on anti-nonspecific adsorption were surveyed (Figure 2b,c). BSA adsorption capacity decreased with increasing DA-PEG_5000_-DA concentration and modification time possibly because of the increase in DA-PEG_5000_-DA loading, and the strength of the steric hindrance effect increased. However, when the DA-PEG_5000_-DA loading reached a certain value, anti-nonspecific performance did not change. Thus, the optimal synthesis conditions of AmZPP_x_ were as follows: molecular weight of PEG midblock, 5000; DA-PEG_5000_-DA concentration, 0.2 mM; and contact time, 30 min.

### 2.2. Characterization

**Figure 3:**
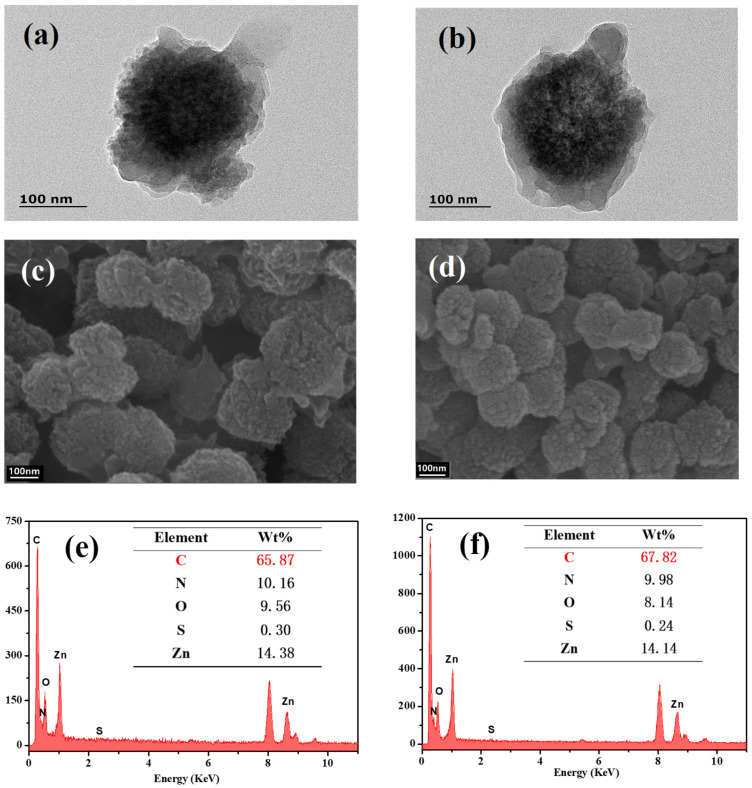
TEM, SEM and EDS images of AmZP (**a**, **c** and **e**, respectively) and AmZPP_5000_ (**b**, **d** and **f**, respectively).

The morphology of the synthesized materials was characterized by transmission electron microscopy (TEM) and scanning electron microscopy (SEM). As shown in Figure 3, AmZP and AmZPP_5000_ had rough surfaces with diameters ranging from 100 nm to 200 nm, indicating that the polymer modification process did not significantly change the particle size. The TEM images indicated a distinct core–shell structure. Energy-dispersive spectroscopy (EDS) characterization of AmZP and AmZPP_5000_ revealed the presence of ZIF-8 (Zn and N elements) and protein (S element), as shown in Figure 3e,f. The proportion of C element in AmZPP_5000_ increased relative to that in AmZP, whereas the proportion of other elements decreased, illustrating successful modification by DA-PEG_5000_-DA. 

The Fourier-transform infrared (FT-IR) spectra of AmZP and AmZPP_5000_ are shown in Figure 3. The wave numbers between 1600–1700 cm^−1^ and 1500–1580 cm^−1^ were attributed to amide I and amid II bands, respectively, and indicated the existence of enzymes [48]. The peak at ~1200 cm^−1^ represented the –OH vibration of phenol compounds, and indicated the presentation of PDA [46]. Moreover, a new absorption peak was detected at 841 cm^−1^ due to the bending vibration of –C–O–C–. This result indicated the successful combination of DA-PEG_5000_-PEG.

**Figure 4:**
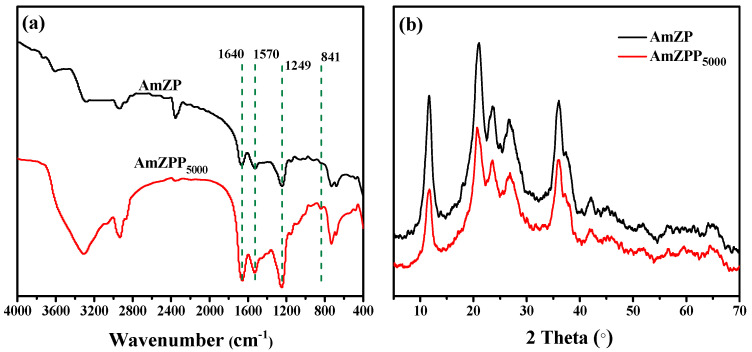
FT-IR (**a**) and XRD (**b**) spectra of AmZP and AmZPP_5000_.

The X-ray diffraction (XRD) patterns for AmZP and AmZPP_5000_ are shown in Figure 4. Both materials presented the same characteristic peaks as ZIF-8 [48], but the peaks were diffused, indicating that the amorphous structures in the materials with short-range order and long-range disorder stacking. Mesopores in amorphous MOFs can make encapsulated enzymes have a higher activity than crystalline MOFs [49].

**Figure 5:**
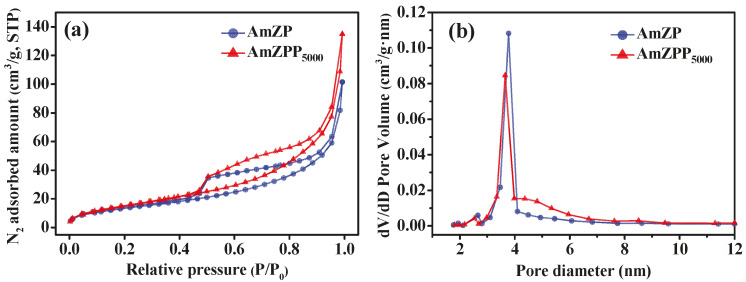
BET curves (**a**) and diagram pore sizes distribution (**b**) of AmZP and AmZPP_5000_.

The specific surface areas and pore structures of AmZP and AmZPP_5000_ were characterized by N_2_ adsorption–desorption experiments. The Brunauer–Emmett–Teller (BET) curves displayed in Figure 5a exhibited a type IV adsorption isotherm with an H3 loop and indicated that both materials were mesoporous [50]. In addition, AmZP and AmZPP_5000_ did not reach adsorption equilibrium when the relative pressure was close to the saturation pressure, indicating that the measured materials consisted of mesoporous slits. Based on the Barrett–Joyner–Halenda model (Figure 5b), the distribution diagram pore sizes of the two samples revealed an increase in 4–8 nm pores after DA-PEG_5000_–PEG modification on AmZP, whereas the pore sizes were still mostly distributed in the 2–4 nm range. The specific surface area of AmZPP_5000_ increased slightly from 51.16 m^2^/g to 60.37 m^2^/g compared with that of AmZP. These results suggested that DA-PEG_5000_–PEG attached to AmZP surface via the “grafting to” strategy had no effect on the main structure of the material. 

### 2.3. Specific Adsorption of AmZPP_5000_

**Figure 6:**
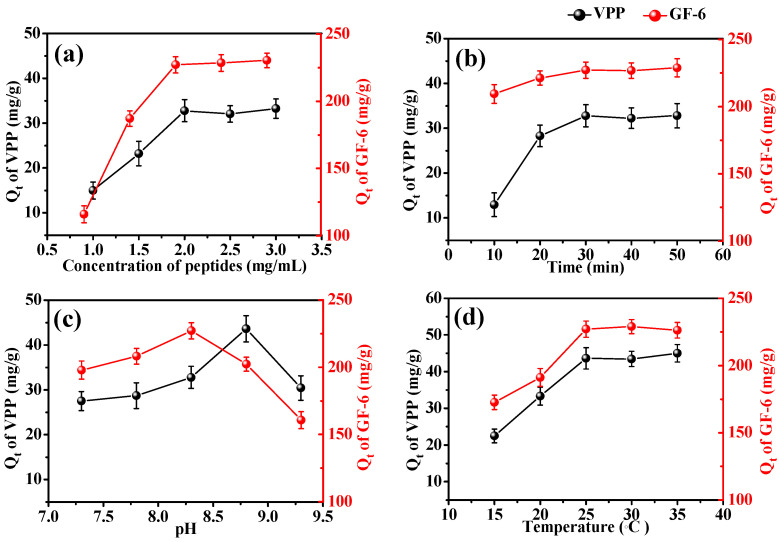
Effects of peptides concentration (**a**), time (**b**), pH (**c**) and temperature (**d**) on the adsorption of AmZPP_5000_ on VPP and GF-6.

The specific adsorption analysis (Figure 6a) revealed that the adsorption capabilities of VPP and GF-6 first increased with peptide concentration and then remained unchanged after the maximum adsorption capability was reached. The mass transfer rate can be improved by increasing the concentration. Adsorption capacity is affected by the amount of encapsulated enzyme, cannot be increased indefinitely, and will not change after adsorption saturation is reached. Moreover, the adsorption capacity of GF-6 is higher than that of VPP because GF-6 is a noncompetitive inhibitor with multiple binding sites on ACE [45] and VPP is a competitive inhibitor that mainly binds to active sites of enzymes [43]. 

At initial VPP and GF-6 concentrations of 2 mg/mL, the relationship between the adsorption capacity of AmZPP_5000_ and time was measured (Figure 6b). At 0–30 min, the adsorption capacity gradually increased with time because the peptides did not immediately reach the adsorption sites. At 30–60 min, the adsorption capacity remained unchanged, indicating that the binding sites of AmZPP_5000_ reached saturation.

Figure 6c,d show the effects of pH and temperature on the adsorption of AmZPP_5000_ on VPP and GF-6. The adsorption amount varied with pH and temperature because the adsorption of the two peptides was based on the specific interaction with ACE. The mass transfer rate and structure of the peptides change under experimental pH and temperature conditions, and thus change the adsorption behavior. 

### 2.4. Adsorption Kinetics of AmZPP_5000_ for VPP and GF-6

The pseudo-first order (PFO) and pseudo-second order (PSO) and intraparticle diffusion (IPD) models [51,52] were employed to fit the adsorption kinetic data obtained under the following conditions: temperature, 25 °C; pH, 8.3 (for GF-6) or 8.8 (for VPP); AmZPP_5000_, 10 mg; peptides concentration, 2 mg/mL.

The fitting equation of the PFO model is expressed as follows:

(1) $$Q_{t}=Q_{e}\left(1-e^{-k_{1}t}\right)$$

The fitting equation of the PSO model is expressed as follows:

(2) $$Q_{t}=\frac{k_{2}{Q_{e}}^{2}t}{1+k_{2}Q_{e}t}$$

The fitting equation of the IPD model is expressed as follows:

(3) $$Q_{t}=k_{id}t^{\frac{1}{2}}+C$$

where *Q_t_* (mg/g) and *Q_e_* (mg/g) are the adsorption capacities of peptides at a given time (*t*) and at equilibrium, respectively; *k*_1_ and *k*_2_ are the rate constants of the PFO and PSO kinetic models for adsorption, respectively; *k_id_* is the intraparticle diffusion constants; and *C* is the effect of external boundary diffusion layer on adsorption process.

**Figure 7:**
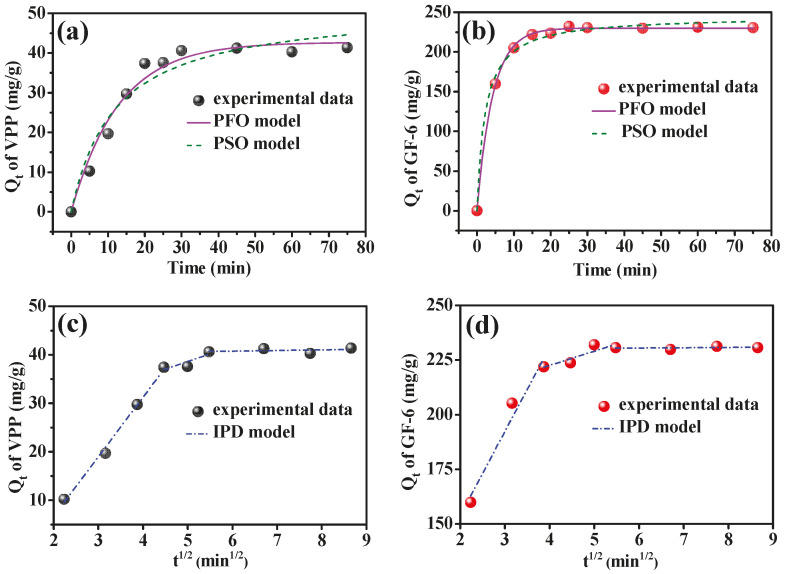
Adsorption kinetics of AmZPP_5000_ for VPP (**a**,**c**) and GF-6 (**b**,**d**) fitting by PFO, PSO and IPD models.

The fitting results are shown in Figure 7, and the adsorption parameters are displayed in Table 1. As expected, adsorption increased with contact time, and VPP and GF-6 achieved adsorption equilibrium after 30 min. The *Q_e,cal_* value obtained from the PFO model was closer to the experimental *Q_e,exp_* than the *Q_e,cal_* from the PSO model, and the R^2^ in PFO was better than that in PSO. The adsorption rate constant of AmZPP_5000_ for VPP (*k*_1_ = 0.0774) was lower than that for GF-6 (*k*_1_ = 0.2319), indicating a slower rate by the former than the latter. Above results suggested that the PFO model is more suitable for describing the adsorption processes of VPP and GF-6 on AmZPP_5000_, which is related to physical adsorption.

The adsorption-rate-determining steps were investigated using the IPD model (Figure 7c,d and Table 2). The IPD fitting results indicated that the data points had three linear parts, suggesting the adsorption of VPP or GF-6 for AmZPP_5000_ was associated with three continuous processes. The first step is related to surface diffusion; the second step is the diffusion process within a particle; and the third step is the dynamic equilibrium process of adsorption and desorption. Moreover, the plotted lines did not penetrate the origin (C ≠ 0) and k_id_ in the second step was lower than that in the first step, indicating intraparticle diffusion was the main process but not the only control process.

### 2.5. Adsorption Isotherm of AmZPP_5000_ for VPP and GF-6

Experimental adsorption isotherm data were obtained under the following conditions: temperature, 25 °C; pH, 8.3 (for GF-6) or 8.8 (for VPP); AmZPP5000, 10 mg; contact time, 30 min. The Langmuir, Freundlich and D–R (Dubinin–Radushkevich) isotherm models [53] were used to describe the adsorption processes of VPP and GF-6.

The Langmuir model is represented by the following equation:

(4) $$Q_{e}=\frac{Q_{m}k_{L}C_{e}}{1+k_{L}C_{e}}$$

The Freundlich model is represented by the following equation:

(5) $$Q_{e}=k_{F}C_{e}^{\frac{1}{n}}$$

The D–R adsorption model is represented by the following equation:

(6) $$\ln Q_{e}=lnQ_{m}-B_{DR}\varepsilon_{DR}^{2}\;$$

(7) $$\varepsilon_{DR}=RTln\left(1+\frac{1}{C_{e}}\right)$$

(8) $$E=\frac{1}{\sqrt{2B_{DR}}}$$

where *Q_e_* (mg/g) and *Q_m_* (mg/g) are the equilibrium and theoretical maximum adsorption capacities of peptides, respectively; *k_L_* represents the Langmuir constants; *k_F_* and *n* represent the Freundlich constants; *C_e_* is equilibrium peptide concentration; *B_DR_* is the activity coefficient related to adsorption energy (mol^2^/J^2^); *ε_DR_* is Polanyi potential energy; R is gas constant (8.314 J/mol/K) and *T* is absolute temperature (K); *E* is mean Gibbs free adsorption energy.

**Figure 8:**
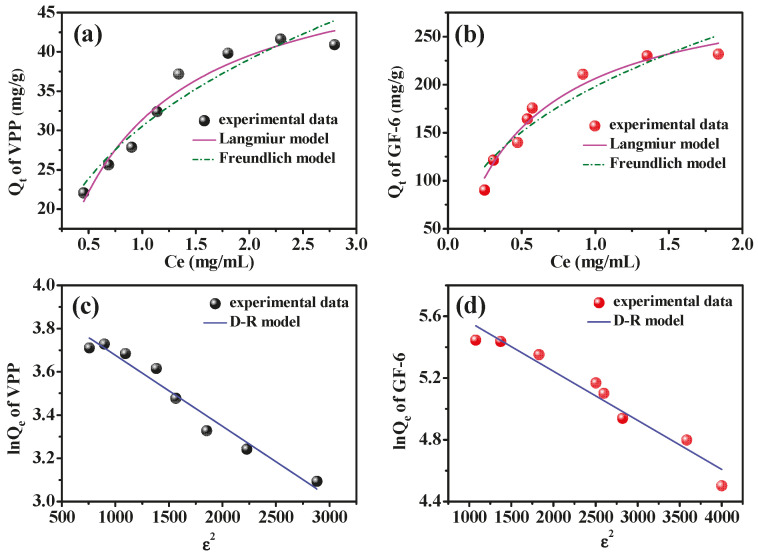
Adsorption isotherm of AmZPP_5000_ for VPP (**a**,**c**) and GF-6 (**b**,**d**) fitting by Langmuir, Freundlich and D–R models.

The results are shown in Figure 8 and Table 3. The adsorption of VPP and GF-6 on AmZPP_5000_ was more consistent with the Langmuir model than with the Freundlich model, suggesting the adsorption was a monolayer process. Moreover, the maximum adsorption capacity *Q_max_* of Langmuir fitting for VPP and GF-6 were 53.44 and 308.78 mg/g, respectively, indicating that AmZPP_5000_ is a promising material for enrichment of ACE inhibitory peptides with excellent adsorption capacity.

The D–R model has no assumption of single-layer, homogeneous adsorption and could be used to distinguish physical adsorption from chemical adsorption. As seen from Figure 8c,d and Table 3, the D–R model fits the experiment data quite well. The *E* values of VPP and GF-6 were 0.552 and 0.561 kJ/mol, respectively. Usually, when *E* < 8.0 kJ/mol, physical adsorption can be considered as the main adsorption process, while chemical adsorption is the main adsorption process when *E* > 8.0 kJ/mol.

Above results indicated the adsorption process of VPP and GF-6 with AmZPP_5000_ was physical monolayer adsorption with a limited number of isolated adsorption sites, which was consistent with previous reports that VPP and GF-6 bonded to ACE primarily via hydrogen bonding and hydrophobic interactions [43,45].

### 2.6. Desorption and Reusability of AmZPP_5000_

**Figure 9:**
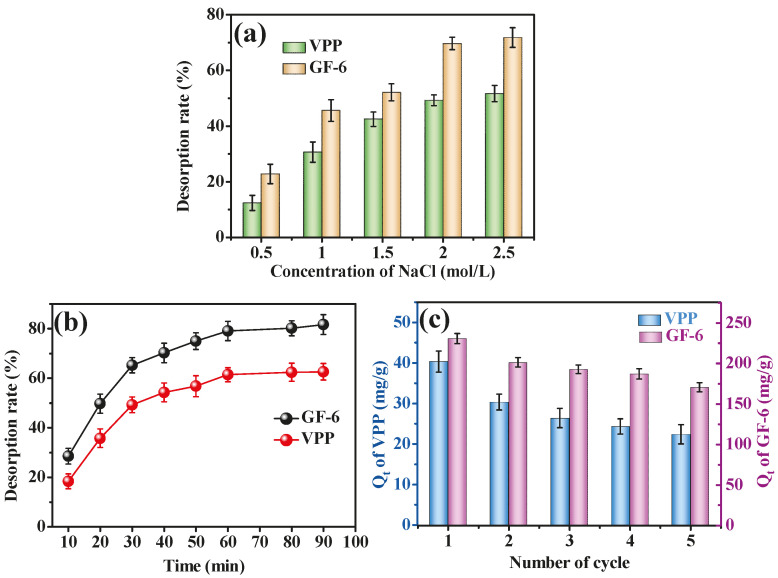
Desorption (**a**,**b**) and reusability (**c**) of AmZPP_5000_.

Desorption and reusability are important indexes for materials’ practical applications. The adsorption of ACE inhibitory peptides and ACE belongs to affinity adsorption, which usually requires a high concentration of NaCl for desorption [23]. As shown in Figure 9a,b, the desorption effect of 2 mol/L NaCl on VPP and GF-6 was significantly higher than 1.5, 1.0 and 0.5 mol/L and reached almost 80% at 1 h desorption time. This result suggested that 2 mol/L NaCl can be used as the desorption agent for the desorption of ACE inhibitory peptides from AmZPP_5000_.

It has been reported that amino acids and protein could destroy the structure of ZIF-8 because of the complexation of amino acids with zinc ions [54]. NaCl could also affect the structure of ZIF-8 [55]. As shown in Table 4, peptides NaCl and BSA could cause slight enzyme release from ACE@mZIF-8. The PDA layer could protect mZIF-8 against agents, making AmZP and AmZPP5000 more stable.

The reusability of AmZPP_5000_ was determined by repeating adsorption–desorption process. As shown in Figure 9c, AmZPP_5000_ had excellent recycling performance after the fifth cycle, where the adsorption capacities of VPP and GF-6 were 22.39 mg/g and 170.58 mg/g, respectively. This decrease might have been caused by the incomplete desorption and the mass loss of the AmZPP_5000_ after washing and during the repeated adsorption–desorption processes.

### 2.7. Recognition Selectivity of AmZPP_5000_

**Figure 10:**
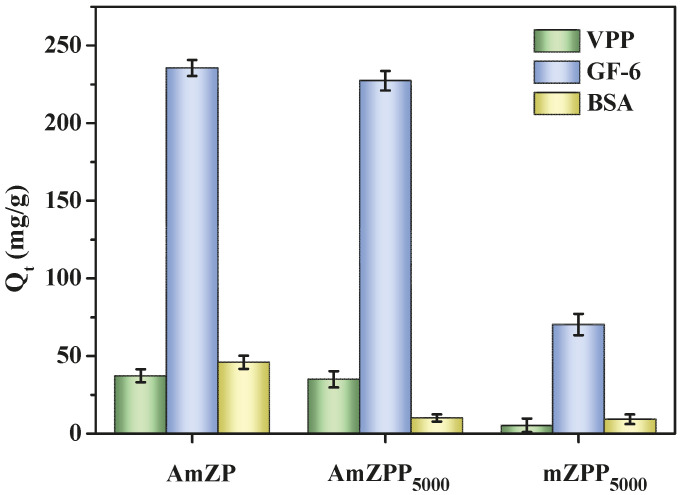
Adsorption data of AmZPP_5000_ in mixed system.

A mixture containing VPP, GF-6, and BSA as the adsorption sample was used in investigating the recognition selectivity of AmZP, AmZPP_5000_ and mZPP_5000_ (without enzyme). The results are illustrated in Figure 10. Obviously, AmZPP_5000_ and AmZP exhibited a higher adsorption capacity for VPP and GF-6 than mZPP_5000_ for the specific interaction between peptides and ACE. All the materials displayed higher adsorption capacities for peptides than BSA. The reason was that BSA mainly bound to the materials’ surfaces because of its diameter (~7 nm) [56], which was larger than the pore size of the materials (2–4 nm). BSA was unable to access the pores. By contrast, the small molecular peptides were transported to the mesopores and thus showed higher adsorption capacities. In the absence of anti-protein adsorption chain segments, the BSA adsorption capacities of AmZPP_5000_ and mZPP_5000_ was lower than that of AmZP. The results showed that the introduction of DA-PEG_5000_-DA polymer loops can indeed improve the recognition selectivity of immobilized ACE.

### 2.8. Application of AmZPP_5000_ in Hydrolysate

**Figure 11:**
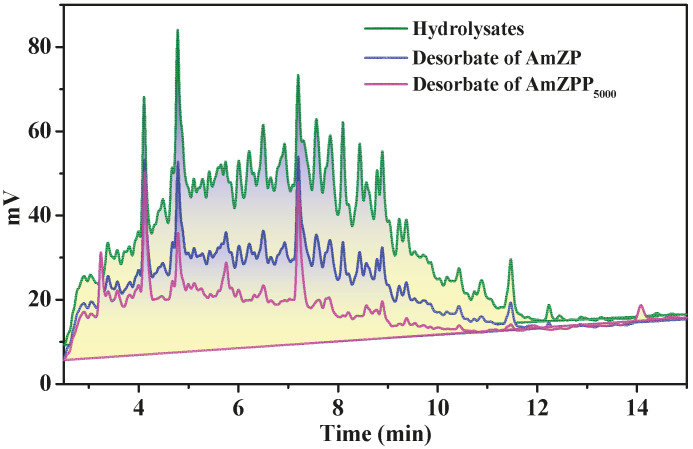
HPLC chromatogram on a Zorbax SB C18 column of samples. Experiments were carried out with a linear gradient of 5–50% acetonitrile in water (containing 0.1% TFA) over 15 min at a flow rate of 1 mL/min.

Based on the above results, AmZPP_5000_ displayed excellent adsorption performance and reusability and provided a possibility for further application. The recognition selectivity of AmZP was compared with that of AmZPP_5000_ by using a hydrolysate (obtained from *Leiognathus brevirostris*) containing ACE inhibitory peptides. The concentrations of proteins in the desorption solutions were determined using UV spectrophotometry. As shown in Figure 11 and Table 5, both materials had screening functions, and the desorbate of AmZPP_5000_ displayed simpler components than that of AmZP and achieved lower IC_50_ value, which indicated a higher ACE inhibitory activity. These results demonstrated that the recognition selectivity of AmZP was successfully enhanced by anchoring polymer loops on the surface, and the AmZPP_5000_ could be used to enriched ACE inhibitory peptides.

## 3. Materials and Methods

### 3.1. Materials

Crude ACE was isolated according to the reported literature [44]. Hippuryl-histidyl-leucine (HHL; ≥99%) and ACE (from rabbit lung) were purchased from Sigma Chemical Co. (St. Louis, MO, USA). 2-MeIM (≥99%), DA (≥99%) and HPLC-grade trifluoroacetic acid were purchased from Shanghai Aladdin Biochemical Technology Co., Ltd. (Shanghai, China). DA-PEGx-DA (x = 2000, 5000 and 10,000) were purchased from Guangzhou Tansh-tech Co., Ltd. (Guangzhou, China). VPP and GF-6 were obtained from GL Biochem (Shanghai) Ltd. (Shanghai, China). HPLC-grade methanol and acetonitrile were purchased from Shanghai Macklin Biochemical Co., Ltd. (Shanghai, China). All the other reagents were of analytical grade and purchased from XiLong Science (Guangdong, China).

### 3.2. Preparation of AmZP

For the synthesis of AmZP, a 100 mL beaker containing Zn (NO3)_2_·6 H_2_O (0.0297 g), 2-MeIM (0.0657 g), 5 mL of crude ACE solution (protein concentration of 4 mg/mL and specific activity of 0.013 U/mg protein), and 30 mL of 0.1 M borate buffer solution (BBS, pH 7.3) was incubated at 30 °C for 30 min. The prepared ACE@mZIF-8 was collected by centrifugation at 8000 rpm for 10 min and washed with distilled water three times. Then, ACE@mZIF-8 was dispersed in 10 mL of 50 mM Tris-HCl (pH = 8.5), and 10 mg of DA was added. The mixture was gently agitated for 4 h at room temperature. The obtained AmZP was washed several times with deionized water and then vacuum freeze-dried.

### 3.3. Surface Modification with DA-PEG_x_-DA

The prepared AmZP (100 mg) was dispersed in 20 mL of distilled water, and 20 mg of DA-PEG_x_-DA (x = 2000, 5000, 10,000) was added. The mixture was mildly stirred for 1 h at room temperature. Then, the product AmZPP_x_ was collected by centrifugation and washed with distilled water three times.

### 3.4. Characterization

SEM images were recorded using TESCAN MIRA LMS (Brno, Czech Republic) at a voltage of 3 kV. TEM images and EDS analysis results were obtained using JEOL JEM-F200 (Tokyo, Japan) at an operating voltage of 200 kV. FT-IR spectra were tested at a wavenumber range of 400–4000 cm^−1^ with a Nicolet IS10 (Waltham, MA, USA). XRD analysis was carried out using Mini Flex600 (Cu-Kα radiation), and the diffraction patterns were obtained from 5° to 70° with a scanning rate of 10°/min at 45 kV and 15 mA. Specific surface areas were measured at 77.35 K with the BET method in a relative pressure range (P/P_0_) of 0.05–1. The amount of protein encapsulated in the material was determined by the Bradford method.

### 3.5. ACE Activity and Inhibitory Activity Assay

The digestion of HHL into hippuric acid (HA) and His-Leu (HL) using free or immobilized ACE was performed, and the activity of the enzyme was evaluated. Free ACE solution (protein concentration of 1 mg/mL) or immobilized ACE suspension (mass concentration of 5 mg/mL) were prepared in 0.1M BBS (containing 0.3 M NaCl, pH 8.3). Approximately 60 µL of the sample and 100 µL of 0.1M BBS (containing 0.3 M NaCl, pH 8.3) were incubated at 37 °C for 10 min. Then, 40 µL of 5 mM HHL (in BBS) was added and reacted for 15 min. The reaction was stopped by adding 100 µL of 1 M HCl. The activities of the free and immobilized ACE were measured by monitoring the released HA with the HPLC method.

ACE inhibitory activity was evaluated according to the above description with some modification. ACE solution (0.001 U/mL) was prepared using commercial ACE and the sample was used instead of the BBS buffer. The ACE inhibitory activity was calculated by the following equation [23]:

(9) $$ACE\;inhibition\;rate=\frac{A_{0}-A_{1}}{A_{0}}\times 100\%\;\;\;\;$$

where *A*_0_ and *A*_1_ are the peak areas of HA in the control group (without inhibitory peptides) and the sample (containing inhibitory peptides), respectively. IC_50_ is defined as the concentration of an inhibitor when half of the enzyme activity is suppressed.

### 3.6. Concentration of VPP and GF-6 Analysis

An RP-HPLC column (Zorbax SB-C18, 4.6 mm × 150 mm, Agilent, Palo Alto, CA, USA) was applied to measure the concentration of VPP and GF-6 with the solvent system consisting of 0.1% trifluoroacetic (in water, solvent A) and acetonitrile (containing 0.1% trifluoroacetic, solvent B). For VPP, a gradient of 5–15% solvent B over 15 min at a flow rate of 1 mL/min was used, while a gradient of 15–35% solvent B over 15 min at the same flow rate for GF-6. During the analysis process, the detector wavelength was set as 220 nm.

### 3.7. Specific/Nonspecific Adsorption Tests

BSA was used as a protein model for nonspecific adsorption. In this work, two known ACE inhibitory peptides, VPP (competitive inhibitory peptides of ACE) and GF-6 (noncompetitive inhibitory peptides of ACE), were selected for specific adsorption experiments.

The solutions of protein or peptides with different concentrations in 0.1 M BBS were prepared. For adsorption, the adsorbent and peptide solutions were mixed and shaken for a specified time. Then, the mixtures were centrifuged, and the concentrations of the peptides were detected via HPLC.

The adsorption amount of the materials was calculated as follows:

(10) $$Q_{t}=\frac{\left(C_{0}-C_{t}\right)\times V}{m}$$

(11) $$Q_{e}=\frac{\left(C_{0}-C_{e}\right)\times V}{m}$$

where *Q_t_* and *Q_e_* are the adsorption amounts of peptides (mg/g) at time *t* and at equilibrium, respectively; *C*_0_, *C*, and *Ce* are the initial concentration, concentration at time *t*, and equilibrium concentration of a peptide solution (mg/mL), respectively; *V* is the volume of peptide solutions (mL); and *m* is the weight (g) of the immobilized enzyme.

### 3.8. Studies of Stability

The activity of ACE@mZIF-8, AmZP and AmZPP_5000_ was monitored after incubation with 0.5 mg/mL BSA or 2 M NaCl at room temperature for 30 min. Because of the inhibitory effect of peptides on ACE, the stability of the material cannot be determined by measuring the activity after incubation with 2mg/mL VPP and GF-6. Therefore, the stability of the material was determined by measuring the protein concentration of the supernatant via the Bradford method.

### 3.9. Application of AmZPP_5000_ in Hydrolysate Samples

The hydrolysates were prepared as follows: The fish meal of *Leiognathus brevirostris* (100 g) in 300 mL of 0.05 M PBS (pH 9.5) was heated in a boiling water bath for 10 min, and cooled to 45 °C. Alcalase was added in an enzyme/protein molar ratio of 4000 U/g for 3 h at 45 °C. Then, 1 mol/L NaOH was used to maintain the pH at 9.5 during hydrolysis. Enzymatic hydrolysis was terminated through inactivation in a boiling water bath for 10 min. The hydrolysate was collected by centrifugation (8000 rpm, 15 min) and then stored at −20 °C.

Approximately 10 mg of AmZPP_5000_ was added to the hydrolysate with a shaking rate of 120 rpm for 30 min. After adsorption, the desorption experiment was performed in 2 M NaCl solution for 30 min at room temperature. The inhibitory activity of the supernatant was determined.

### 3.10. Statistical Analysis

All the experiments were carried out in triplicate, and the results are presented as average ± standard deviations. Statistically significant differences of the collected data were analyzed with one-way ANOVA. Statistical significance was established at *p* < 0.05.

## 4. Conclusions

In this work, the surface of immobilized ACE AmZP was successfully modified by (DA-PEG_x_-DA) via a convenient “grafting to” strategy for the fabrication of a novel material (AmZPP_x_) with high recognition selectivity for ACE inhibitory peptides. AmZPP_5000_ with molecular weight of PEG midblock of 5000 showed the best anti-nonspecific adsorption performance. The specific adsorption capacities of AmZPP_5000_ for VPP (competitive inhibitor for ACE) and GF-6 (noncompetitive inhibitor for ACE) were considerably higher than nonspecific adsorption capacity. Adsorption kinetic and adsorption isotherm studies showed that the adsorption of VPP and GF-6 belongs to physical monolayer adsorption and the diffusion was mainly controlled by intraparticle diffusion. Regarding the contribution of DA-PEGx-DA polymer loops, AmZPP_5000_ presented excellent ability to specifically recognize ACE inhibitory peptides in actual hydrolysates.

Immobilized enzymes are becoming rapid and efficient platforms for screening bioactive components from complex products. We anticipate that capitalizing on the rational design of polymer loops modified on immobilized enzyme surfaces will benefit applications involving separation processes.

## Figures and Tables

**Table 1:** Kinetic parameters for VPP and GF-6 adsorption on AmZPP_5000_.

		PFO Model			PSO Model		
	***Q_e,exp_*** **(mg/g)**	***Q_e,cal_*** **(mg/g)**	***k*_1_**	**R^2^**	***Q_e,cal_*** **(mg/g)**	***k*_2_**	**R^2^**
VPP	40.88	42.68	0.0774	0.9757	51.61	0.00163	0.9472
GF-6	230.37	229.98	0.2319	0.9992	245.29	0.00182	0.9926

**Table 2:** IPD kinetic parameters for VPP and GF-6 adsorption on AmZPP_5000_.

	First Step			Second Step			Third Step
	***K_id_*** **(mg/(g** **∙min^1/2^)**	***C***	**R^2^**	***K_id_*** **(mg/(g** **∙min^1/2^)**	***C***	**R^2^**	
VPP	12.26	−17.84	0.9924	3.134	22.91	0.8777	Balance stage
GF-6	38.49	76.70	0.9326	6.511	196.45	0.7069	

**Table 3:** Adsorption isotherm parameters of AmZPP_5000_ for VPP and GF-6.

	Langmuir Model			Freundlich Model			D–R Model		
	*k_L_* (mL/mg)	*Q_m_* (mg/g)	R^2^	*k_F_*	*n*	R^2^	*Q_m_* (mg/g)	*E* (KJ/mol)	R^2^
VPP	1.4211	53.44	0.9501	30.53	2.816	0.9107	54.86	0.552	0.9556
GF-6	2.0131	308.78	0.9589	198.02	2.544	0.8860	357.70	0.561	0.9441

**Table 4:** Release of ACE from ACE@mZIF, AmZP and AmZPP_5000_ after treatment with different agents for 1 h at 25 °C.

	Protein release (%)		
Agents	ACE@mZIF-8	AmZP	AmZPP_5000_
2 mg/mL VPP	7.32	1.37	1.24
2 mg/mL GF-6	6.21	1.08	1.13
	Enzyme activity release (%)		
2 mol/L NaCl	3.84	0.21	0.20
0.5 mg/mL BSA	1.52	0.00	0.00

**Table 5:** Selective screening of ACE inhibitory peptides from hydrolysates by AmZP and AmZPP_5000_.

Sample	IC_50_ (mg/mL)
Hydrolysates	0.623
Desorbate of AmZP	0.095
Desorbate of AmZPP_5000_	0.034

## Data Availability

Not applicable.

## Sample Availability

Samples of the compounds are available from the authors.
